# Rupture of the ilio-psoas tendon after a total hip arthroplasty: an unusual cause of radio-lucency of the lesser trochanter simulating a malignancy

**DOI:** 10.1186/1749-799X-5-6

**Published:** 2010-02-05

**Authors:** Aditya V Maheshwari, Rajesh Malhotra, Deepak Kumar, J David Pitcher

**Affiliations:** 1 Musculoskeletal Oncology, Department of Orthopaedics, University of Miami Miller School of Medicine, 1400 NW 12th Ave University of Miami Hospital, East Building, #4036 Miami, FL 33136, USA; 2 All India Institute of Medical Sciences, Ansari Nagar, New Delhi, 110029, India; 3 Department of Biomechanics and Movement Science, University of Delaware, Newark, DE 19711, USA

## Abstract

Avulsion fracture or progressive radiolucency of lesser trochanter is considered a pathognomic finding in patients with malignancies. Although surgical release of the iliopsoas tendon may be required during a total hip arthroplasty (THA), there is no literature on spontaneous rupture of the ilio-psoas tendon after a THA causing significant functional impairment. We report here such a case, which developed progressive radiolucency of the lesser trochanter over six years after a THA, simulating a malignancy. The diagnosis was confirmed by MRI. Because of the chronic nature of the lesion, gross retraction of the tendon into the pelvis, and low demand of our patient, he was treated by physiotherapy and gait training. Injury to the ilio-psoas tendon can occur in various steps of the THA and extreme care should be taken to avoid this injury. Prevention during surgery is better, although there are no reports of repair in the THA setting. This condition should be considered in patients who present with progressive radioluceny of the lesser trochanter, especially in the setting of a hip/pelvic surgery. Awareness and earlier recognition of the signs and symptoms of this condition will aid in diagnosis and will direct appropriate management.

## Introduction

Avulsion fracture of lesser trochanter of the femur is a well known entity in children and adolescents [1]. However, its fracture or progressive radiolucency is considered a pathognomic finding in adults with malignancies [2]. Spontaneous rupture of ilio-psoas tendon is rare and has not been described before in the setting of a total hip arthroplasty (THA). We present here such a case who had a spontaneous rupture of the ilio-psoas tendon few days after a THA with subsequent progressive radiolucency of the lesser trochanter, simulating a malignancy. Awareness of this entity would aid in the diagnosis, prevent confusion with malignant disease, and allow appropriate management along with patient reassurance.

## Case report

**Figure 1:**
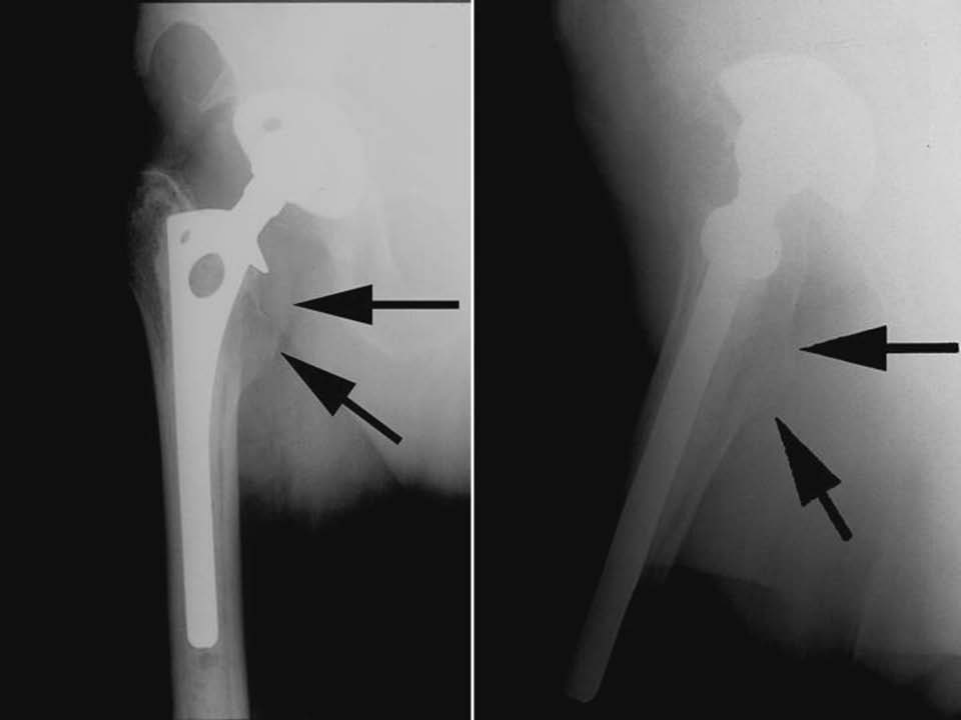
**AP and lateral views of the right hip showing a well fixed hybrid implants with a radio-lucency around the lesser trochanter region (arrows), suggesting disuse atrophy in retrospective**.

A 77-year old, otherwise healthy, sedentary male was referred to the orthopedic oncology service for a progressive radiolucency of the lesser trochanter on radiographs (Fig. 1). He had undergone a hybrid THA for degenerative right hip disease at another institute six years ago but had persistent groin pain after the surgery. During a physiotherapy session at two weeks postoperatively, he felt a sudden increase in groin pain and then a 'pop' while negotiating stairs, and was not able to ambulate independently after that. He stopped his therapy and did not see his primary surgeon for the next six weeks. He was then prescribed further therapy which he did not comply and thereafter had been using an assistive device all the time. His pain gradually improved but he had been having persistent difficulty and weakness while walking on uneven surface, getting in and out of car, getting in and out of bed and negotiating stairs. He denied any prolonged medication or any prior injection in his hip.

**Figure 2:**
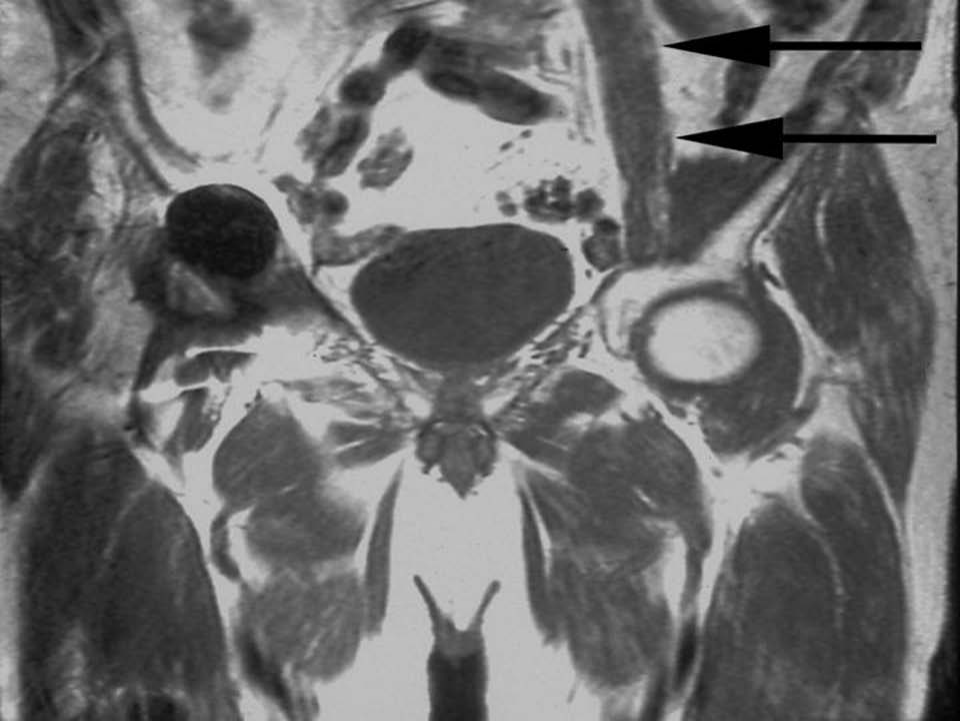
**A coronal T1 MRI showing a normal ilio-psoas tendon on the left side (arrows) but its absence on the right side**.

**Figure 3:**
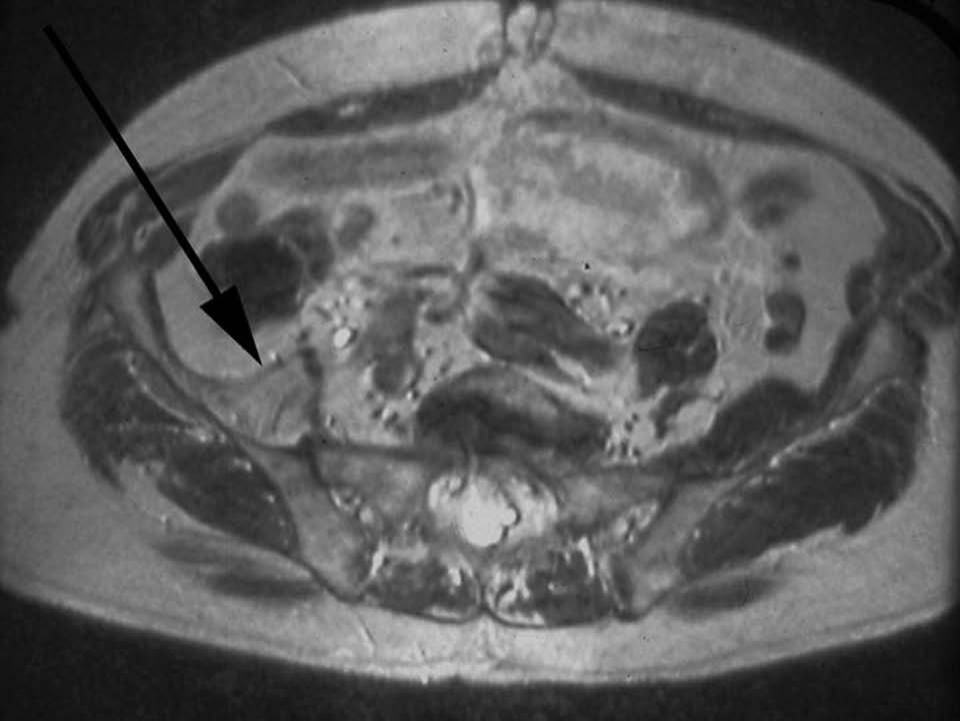
**An axial T2 MRI showing the fatty atrophy and retraction of the right ilio-psoas tendon (arrow) all the way to the level of the sacro-iliac joint**.

On examination, he ambulated with a single crutch. Active straight leg raise was not sustainable. Seated hip flexion was graded as 3/5. There was no tenderness or palpable mass in the groin. Distal neuro-vascular status was intact. The previous surgeons did not recall any intraoperative complication or surgical release of ilio-psoas tendon. Radiographs were not suggestive of implant loosening, malpositioning, osteolysis or wear. Although radiolucency is common in Gruen Zones 7 and 14 after cemented THA [3], it was progressive in this case as compared to previous radiographs and the contralateral side. A Magnetic Resonance Imaging (MRI) showed no lesion in or around the lesser trochanter. Instead it revealed a chronic ruptured ilio-psoas tendon with retraction into the pelvis without residual tendon on the lesser trochanter (Figs. 2 and 3). Laboratory work-up was uneventful. A diagnosis of chronic ilio-psoas tendon rupture with disuse osteopenia of lesser trochanter was made.

Because of the chronic nature of the lesion, gross retraction of the tendon into the pelvis, and low demand of our patient, he was treated by physiotherapy and gait training. At four years follow-up and almost 10 years after his THA, he still uses a cane for outdoor ambulation. Otherwise his medical history has been uneventful with no evidence of any malignancy.

## Discussion

The differential diagnosis of groin pain after a THA includes sepsis, aseptic loosening of components, wear and osteolysis, heterotopic ossification, fracture, neurologic, vascular or abdominal pathology, referred pain from the spine or knee, as well soft tissue irritation such as ilio-psoas tendonitis and synovitis [4-8]. Although ilio-psoas tendonitis has been described in sports medicine and radiology literature [9-12], it is now been increasingly recognized and reported as a cause of groin pain after a THA [4-8]. The incidence of ilio-psoas tendonitis has been reported as high as 4.3% (9 of 206 THAs) [8]. However, postoperative rupture of the ilio-psoas tendon after a THA causing significant functional impairment has not been documented before. Moreover, the progressive radiolucency of the greater trochanter due to disuse raised a suspicion of an underlying malignant process.

Pain specific to ilio-psoas irritation include activities like straight leg raise and resisted hip flexion, passive hyperextension and manifest in day to day life as ascending stairs, lifting the operative leg into the bed, lifting the leg in and out of the car (requiring use of a hand for support) and walking on uneven surface [4-8]. This differentiates it from component loosening, which can cause pain with any weight bearing. The cause has been linked to irritation of the tendon due an anteriorly protruded cup, in cases of a lateralized, oversized or retroverted cup, especially with capsulectomy, protruding screws in the pelvic cavity, overhanging and protruding cement, and also in cases with limb lengthening or an increase in the offset. Although a local anesthetic injection may provide temporary relief and aid in diagnosis, release of the ilio-psoas tendon has been consistently shown to alleviate the symptoms, but component revision may be required in some cases.

An acute rupture of the tendon may manifest as pain in the groin with exacerbation with both flexion and extension of the hip. A palpable mass along with ecchymosis may be present [11]. In earlier setting, an utrasound can demonstrate bursitis, tendonitis or snapping of the tendon over the overhanging acetabulum margin [12]. Apart from showing the soft tissue swelling, a Computed Tomography is also helpful to rule out component malpositiong [6,7]. Although MRI is the most sensitive study to assess the tendon, its role in a THA setting has been traditionally limited due to the artifact generated by metallic implants. However, with modification of pulse sequence with the help of commercially available software, MRI is emerging as an effective tool for assessment of periprosthetic soft tissues, osteolysis and particle disease [13,14]. Axial MR provide the most useful images for diagnosis and in acute cases will show proximal muscle swelling and edema, thickening and interruption of the tendon with an otherwise empty fluid-filled distal tendon sheath [11]. Chronic cases will show muscle atrophy with fatty degeneration.

Although no CT was obtained in our case, radiographs did not show any component malpositioning. Ilio-psoas tendonitis has been shown even in the absence of impingement [7]. However the presence of persisting groin pain with an acute rupture during therapy (loading of the tendon) does indicate a peri-operative injury to the tendon. The ilio-psoas tendon may be injured at the time of exposure, at the time of dislocation, at the time of neck osteotomy, or even at the time of femoral preparation. Moreover, we still do not know the effect of local steroid injection in the tendon as these injections are commonly used peroperatively for postoperative pain management.

There is only one more report of spontaneous atraumatic rupture of distal ilio-psoas tendon in two patients (without arthroplasty); however their medical history was complicated by rheumatoid arthritis, diffuse non-myelinating polyneuropathy, Parkinsonism, Vitamin B-12 deficiency, osteoporosis and Alzheimer's disease [11]. Iliacus muscle injury and resulting hematoma causing femoral nerve palsy has also been described after abdominal extension exercises [15], and also after both cemented and cementless THA, where medial wall has been perforated, especially in patients on anticoagulation therapy [16-19].

## Conclusion

In conclusion, we report a rare instance of rupture of the ilio-psoas tendon after a THA. This condition should be considered in patients who present with progressive radioluceny of the lesser trochanter, especially in the setting of a hip/pelvic surgery. Although weakness of hip flexion has not been reported after tenotomy for ilio-psoas impingement [5,6], our patient had significant functional disability. This may be due to chronic unrecognized tear and lack of physical therapy to train other muscles for hip flexion. Injury to the ilio-psoas tendon can occur in various steps of the THA and extreme care should be taken to avoid this injury. Prevention during surgery is better, although there are no reports of repair in the THA setting. Since ilio-psoas is a postero-medial structure, repair through the most common postero-lateral approach would be difficult because retraction would occur to the medial aspect of the femur and into the inguinal canal. Close postoperative follow-up by the treating physician, and not solely relying on rehabilitative care providers may have identified the rupture in a more timely way. Awareness and earlier recognition of the signs and symptoms of this condition will aid in diagnosis and will direct appropriate management.

## Consent

Written informed consent was obtained from the patient for publication of this case report and the accompanying images. A copy of the written consent is available for review by the Editor-in-Chief of this journal.

## Competing interests

The authors declare that they have no competing interests.

## Authors' contributions

JDP was the surgeon in charge of the patient described with in this report. AVM, DK and RM conducted the literature review and analysed the gathered reports for the described case. AVM, DK and RM composed and wrote the manuscript. All authors read and approved the final manuscript.
